# Modeling rare melanoma subtypes: mechanisms, microenvironments and translational opportunities

**DOI:** 10.3389/fonc.2026.1832600

**Published:** 2026-07-22

**Authors:** Qingxue Meng, Fengxu Yan, Chenchen You, Ruzhong Xu, Taixing Jing, Danyang Ni, Chenyang Hou, Dandan Xu, Shan Liu, Fei Guo, Zhongwu Li, Rensen Ran, Weizheng Liang

**Affiliations:** 1 Hebei Key Laboratory of Systems Biology and Gene Regulation, Technology Department, The First Affiliated Hospital of Hebei North University, Zhangjiakou, Hebei, China; 2 Hebei Key Laboratory of Systems Biology and Gene Regulation, Graduate School of Hebei North University, Zhangjiakou, Hebei, China; 3 Hebei Key Laboratory of Systems Biology and Gene Regulation, Central Laboratory, The First Affiliated Hospital of Hebei North University, Zhangjiakou, Hebei, China; 4 Institute of Bio-Architecture and Bio-Interactions (IBABI), Shenzhen Medical Academy of Research and Translation (SMART), Shenzhen, China; 5 Hebei Key Laboratory of Systems Biology and Gene Regulation, Department of Surgery, The First Affiliated Hospital of Hebei North University, Zhangjiakou, Hebei, China; 6 Department of Pathology, Peking University Cancer Hospital, Beijing, China; 7 School of Basic Medical Sciences, Hainan Medical University, Haikou, Hainan, China

**Keywords:** animal models, genetic accuracy, immunotherapy, microenvironment simulation, rare melanoma subtypes

## Abstract

Rare melanoma subtypes, such as uveal melanoma, desmoplastic melanoma, mucosal melanoma, and acral melanoma, account for about 10% of all melanomas and exhibit unique genetic backgrounds and immune microenvironments, leading to poor clinical prognosis. Due to their low incidence and limited clinical samples, research and therapeutic strategies for these subtypes remain challenging. This review summarizes the current animal models used for rare melanoma, including spontaneous models, inducible models, genetically engineered models, and xenograft models. Each model has its unique advantages, but also has its own limitations in terms of genetic accuracy, microenvironment simulation, or applicability to immunotherapy research. Future advancements in technology should enhance multi-dimensional simulations of both genetics and the microenvironment, thus promoting mechanistic research and the development of precision therapeutic strategies for rare melanoma.

## 1 Introduction

Melanoma is a malignant tumor arising from the malignant proliferation of melanocytes. Its global incidence has shown a significant upward trend in recent years (1, 2). The tumor predominantly occurs in the skin, and a small number of cases are found in the eye, ear, leptomeninges, gastrointestinal tract, as well as the mucosa of the oral cavity, genitalia and paranasal sinuses (3, 4). Ultraviolet (UV) radiation is the major etiological factor for cutaneous melanoma and contributes to the development of melanomas arising from the iris and some subungual acral sites (5, 6). In contrast, several rare melanoma subtypes, including uveal melanoma (UM), acral melanoma (AM), and mucosal melanoma (MM), generally exhibit few or no canonical UV mutational signatures and are characterized by distinct molecular drivers. Although desmoplastic melanoma (DM) is classified as a subtype of cutaneous melanoma, it possesses unique clinicopathological and molecular features that distinguish it from conventional cutaneous melanoma (6, 7). Together, these uncommon melanoma subtypes account for approximately 10% of all melanoma cases (4). These subtypes differ significantly from common cutaneous melanoma in etiology and clinical behavior, leading to generally poor quality of life and prognosis for patients. Due to their low incidence and limited sample size in clinical studies, their biological behavior remains a challenge in melanoma research (8, 9).

Clinically, rare melanomas exhibit higher invasiveness and therapeutic resistance, with a significantly worse prognosis than cutaneous subtypes (10). For instance, MM exhibits relatively low response rates to immune checkpoint inhibitors (ICIs), partly due to impaired IFN-γ signaling within the tumor microenvironment (11). In contrast, although local UM can often be effectively controlled by radiotherapy (12), patients with metastatic UM, particularly those with liver metastases, generally show limited responses to systemic immunotherapy (13). Recent studies have demonstrated that tumor-derived factors, including VGF, can activate hepatic stellate cells and promote the establishment of an immunosuppressive hepatic microenvironment that facilitates immune evasion and therapeutic resistance (13). Moreover, while tebentafusp has improved clinical outcomes in HLA-A*02:01-positive patients with metastatic UM (14), precision therapeutic strategies for rare melanoma subtypes remain inadequate because of limited clinical samples and an incomplete understanding of their molecular mechanisms (11, 13). Most rare subtypes have an extremely low incidence (e.g., the incidence of nail bed melanoma is only 0.1 per 100,000 population), making it difficult to conduct large-scale clinical trials; their unique microenvironmental characteristics (such as mucosal immunosuppression and intraocular immune privilege) further increase the difficulty of mechanistic research (15).

Animal models play a critical role in the progression of melanoma research, driving the translation of basic research to clinical practice. The establishment of appropriate animal models can effectively simulate the occurrence and development of rare melanoma and the therapeutic responses to various treatments, facilitating in-depth studies on the molecular mechanisms of the disease, screening potential therapeutic targets, and evaluating the efficacy of novel drugs (16). Current animal models used in rare melanoma research can be broadly categorized into spontaneous animal models, inducible animal models, genetically engineered animal models, and xenograft models. Each model captures distinct aspects of tumor biology and therefore serves different experimental purposes, ranging from studies of tumor initiation and progression to investigations of metastasis, tumor–host interactions, and therapeutic response (17). However, restricted by current technical limitations and insufficient understanding of the disease, existing animal models still have many limitations in recapitulating the characteristics of human rare melanoma. Some models fail to precisely replicate the interaction between the tumor microenvironment and the immune system, leading to discrepancies between experimental results and clinical reality (18, 19). Moreover, a major unmet challenge across rare melanoma subtypes is the lack of models that can simultaneously preserve tumor-specific genetic alterations, organ-specific microenvironments, metastatic evolution, and functional immune responses. This limitation is particularly evident in studies of liver metastasis in UM, mucosal tissue-specific biology in MM, and tumor–stroma interactions in AM and DM (17, 20). Emerging technologies, including humanized mouse models, CRISPR-based genetically engineered models, patient-derived organoid–xenograft platforms, tissue-engineering approaches, and spatial multi-omics analyses, are expected to address these challenges and facilitate the development of next-generation rare melanoma models (17, 20, 21). Therefore, systematically sorting out the types of existing animal models and comprehensively summarizing their advantages and disadvantages are of great significance for optimizing model construction methods and conducting innovative research.

This review systematically evaluates the establishment methods, application scenarios and limitations of animal models for rare melanoma, with a focus on the specific requirements of each subtype. To provide a comprehensive overview of animal models used in rare melanoma research, a literature search was conducted in PubMed, Web of Science, and Scopus databases up to December 1980 using combinations of keywords including “rare melanoma”, “uveal melanoma”, “mucosal melanoma”, “acral melanoma”, “desmoplastic melanoma”, and “animal model”. Relevant studies focusing on the development, characterization, and application of animal models were selected and reviewed.

## 2 Animal models of uveal melanoma

### 2.1 Biological characteristics of uveal melanoma

UM exhibits a highly specific genetic landscape. Activating mutations at the Q209 locus of the *GNAQ* or *GNA11* gene represent the most central driver event, which persistently activates the downstream MAPK and YAP/TAZ signaling pathways, promoting the abnormal proliferation and transformation of melanocytes (22–24).Based on distinct secondary mutations, UM can be categorized into high-risk and low-risk subtypes: inactivating mutations of the *BAP1* gene are closely associated with a high metastatic risk, whereas tumors harboring *SF3B1* or *EIF1AX* mutations show relatively low metastatic potential (25, 26). This unique genetic profile serves as the core target for establishing UM animal models and a key metric for evaluating model fidelity.

As an immune-privileged site, the eye maintains immune tolerance through multiple mechanisms, such as the expression of Fas ligand (FasL) to induce immune cell apoptosis and the secretion of immunosuppressive factors (e.g., TGF-β, IL-10) (27, 28). UM cells can exploit this microenvironmental feature to evade host immune surveillance, while establishing a tumor microenvironment characterized by immunosuppression—this is also a major reason for the low response rate of immunotherapy in UM (29, 30). Therefore, an ideal UM animal model should recapitulate the unique characteristics of intraocular immune privilege as closely as possible.

**Figure 1:**
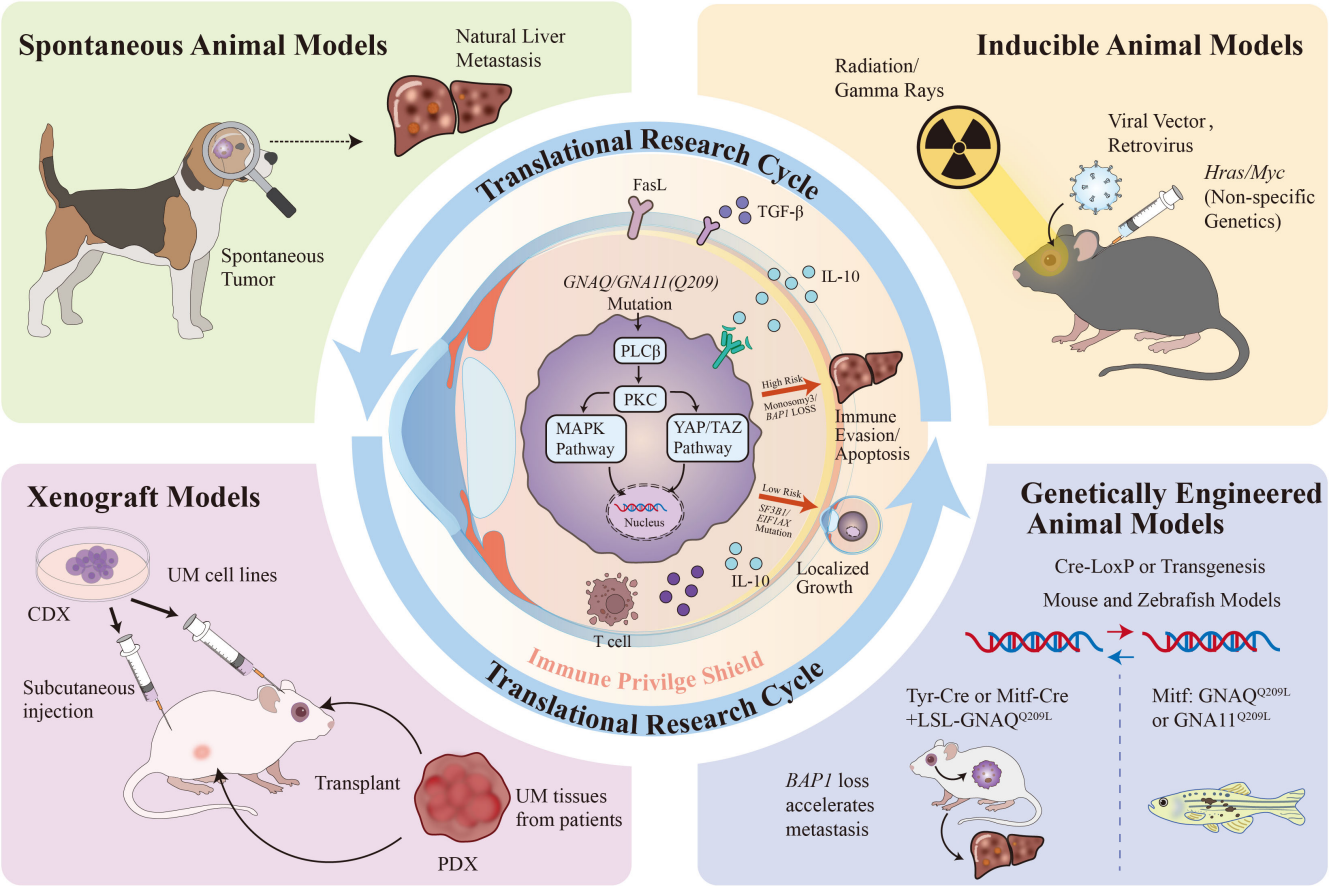
Pathogenesis and translational research models of UM. The central “Immune Privilege Shield” highlights the core pathogenesis, where *GNAQ/GNA11* (Q209) mutations drive oncogenic signaling via PLCβ/PKC to activate the MAPK and YAP/TAZ pathways. The disease course is stratified into high-risk phenotypes characterized by Monosomy 3 and *BAP1* loss, leading to immune evasion and metastasis, versus low-risk phenotypes associated with *SF3B1/EIF1AX* mutations resulting in localized growth. Crucially, the tumor maintains an immunosuppressive microenvironment by secreting FasL, TGF-β, and IL-10 to inhibit T-cell function. Surrounding this mechanistic core are four distinct modeling platforms: Spontaneous Animal Models, utilizing canine subjects that develop natural tumors and liver metastases to study spontaneous progression; Inducible Animal Models, which employ radiation or viral vectors (retroviruses carrying *HRAS/MYC*) to induce tumorigenesis; Xenograft Models, including both CDX via subcutaneous injection of UM cell lines and PDX using direct tissue transplantation; and Genetically Engineered Animal Models (GEMMs), which leverage Cre-LoxP or transgenic systems (e.g., Tyr-Cre or Mitf-Cre combined with LSL-GNAQ) to demonstrate how specific genetic alterations, such as *BAP1* loss, accelerate metastatic potential.

UM metastasis demonstrates striking organ specificity, with the liver being the predominant metastatic target. The underlying mechanisms are closely related to chemokines secreted by tumor cells (e.g., VEGF, VGF), cell adhesion molecules in the liver microenvironment, and the recruitment of immunosuppressive cells (31, 32). Simulating the liver metastatic process of UM is crucial for dissecting metastatic mechanisms and developing anti-metastatic therapeutic strategies, and also represents a key direction to be breakthrough in UM animal models (**Figure 1**).

### 2.2 Spontaneous animal models

Spontaneous models refer to animal models that spontaneously develop UM through natural mutation or genetic screening. Their core advantage is the ability to simulate the natural occurrence and progression of tumors, retaining the complete interaction between the tumor microenvironment and the host immune system (33).

Dogs are currently the most commonly used spontaneous animal models for UM research. The incidence of UM in dogs is relatively higher compared with other spontaneous UM animal models, and they share high similarity with human UM in clinical characteristics, histopathology, and genetic features. For example, canine UM also harbors *GNAQ*/*GNA11* mutations, with liver metastasis as the main metastatic pattern, and abnormal BAP1 protein expression can be observed in some cases (Note: Inactivating *BAP1* gene mutations characteristic of human UM are rarely found in canine UM). In addition, as large mammals, dogs have ocular anatomical structures more similar to humans, which can more truly reflect the invasive process of tumor growth on ocular tissues (34–36). This model has been used to evaluate the efficacy of radiotherapy, local chemotherapy, and immunotherapy, providing important references for the optimization of clinical protocols.

Besides dogs, other mammals such as miniature pigs have also been reported to spontaneously develop UM, but only in case reports (33). Due to their extremely low incidence, high feeding costs, and poor model stability, they have not been widely applied. The main limitations of spontaneous models include a long tumorigenesis cycle (usually 2–5 years for canine UM), large individual differences, and difficulty in conducting precise research on specific genetic targets, which restricts their application in mechanism dissection. Therefore, this type of animal model will not be further described for other subtypes of rare melanoma.

### 2.3 Inducible animal models

Inducible models generate UM in animals through physical, chemical, or viral methods, featuring short construction cycles and low costs, which makes them suitable for large-scale screening studies.

Common physical induction methods include local ocular irradiation (e.g., γ-rays), which induces melanocyte malignant transformation by damaging DNA. For instance, some studies have successfully induced uveal melanoma-like lesions by irradiating the eyes of rats with γ-rays (19, 37). This model can be used to study the molecular mechanisms of radiation-induced tumorigenesis, but the induced tumors have significant differences in genetic characteristics from human UM. Viral-mediated induction methods mainly use retroviruses and lentiviruses to introduce universal oncogenes (e.g., *Hras*, *Myc*, not characteristic driver genes of human UM) into intraocular melanocytes, thereby triggering tumorigenesis (38–40).

However, a significant limitation of inducible models is the strong randomness of the tumorigenic process, making it difficult to accurately simulate the specific genetic background of human UM (e.g., *GNAQ*/*GNA11* driver mutations). Additionally, the induced tumors have great differences in pathological characteristics and metastatic patterns from human UM. Therefore, these models are more suitable for studying the macroscopic mechanisms of tumorigenesis rather than evaluating precise targeted therapies.

### 2.4 Genetically engineered animal models

Genetically engineered models accurately simulate the key genetic mutations of UM in animals via transgenesis, gene knockout, or gene-editing technologies. With high genetic fidelity and strong reproducibility, they represent the core models for current mechanistic studies of UM. Given the central driver role of *GNAQ*/*GNA11* mutations in UM, researchers have successfully established UM models by specifically expressing mutant *GNAQ* or *GNA11* in murine intraocular uveal melanocytes (the major cell-of-origin of UM) under the control of tissue-specific promoters. For instance, driving the expression of the *GNAQ^Q209L^* mutant in murine intraocular uveal melanocytes using Tyrosinase or MITF tissue-specific promoters can induce tumors with pathological features resembling human UM, and some models exhibit signs of liver metastasis. These models not only recapitulate the oncogenic process of UM but also help dissect the regulatory mechanisms of the signaling pathways downstream of *GNAQ*/*GNA11*, providing an ideal *in vivo* evaluation platform for targeted drug development (23, 41–43). To model high-risk UM, researchers have further developed genetically engineered models with combined mutations. For example, knocking out the *BAP1* gene on the basis of *GNAQ* mutation markedly enhances tumor invasiveness and liver metastatic potential (41, 44). This model recapitulates the clinical characteristics of high-risk human UM and serves as a valuable tool for studying *BAP1*-mediated metastatic mechanisms (39). Furthermore, models harboring *SF3B1* or *EIF1AX* mutations are currently under construction, which are expected to further refine the genetic simulation system of UM (39, 45).

In addition to mouse models, zebrafish have emerged as a valuable vertebrate platform for studying UM. Owing to their optical transparency during early development, high fecundity, low maintenance cost, and amenability to rapid genetic manipulation, zebrafish provide unique advantages for investigating tumor initiation, dissemination, and therapeutic responses *in vivo (*17, 46). Several transgenic zebrafish models have been generated by expressing oncogenic *GNAQ^Q209L^* or *GNA11^Q209L^* under melanocyte-specific promoters, resulting in the development of pigmented intraocular lesions that recapitulate key molecular features of human UM (46). Moreover, zebrafish models carrying combined alterations in *GNAQ/GNA11* and *BAP1* have been used to study tumor progression and metastatic behavior, providing insights into the cooperative effects of driver mutations and tumor suppressor loss (47). Compared with murine models, zebrafish enable faster model generation, shorter tumor latency, and higher-throughput genetic and drug screening, making them particularly suitable for functional validation studies and early-stage therapeutic discovery (17, 46). In addition, zebrafish xenograft systems allow real-time visualization of tumor cell migration and metastatic dissemination at single-cell resolution, further expanding their utility in UM research (17, 47).

Despite their utility, genetically engineered UM models still have several limitations. Murine models may not fully recapitulate the anatomical and physiological characteristics of the human uveal tract, whereas zebrafish models differ substantially from humans in ocular structure and immune system composition. In addition, although zebrafish facilitate rapid genetic manipulation and high-throughput screening, they cannot completely reproduce the complexity of human metastatic disease, particularly liver-specific immune interactions. Therefore, no single genetically engineered model can fully capture the genetic heterogeneity, metastatic progression, and tumor microenvironment of human UM, highlighting the need for complementary use of multiple model systems.

### 2.5 Xenograft models

Xenograft models are established by transplanting human UM cell lines or tumor tissues into immunodeficient animals, enabling rapid construction of tumor models. As common tools for clinical translational research, they mainly include two types: cell line-derived xenograft (CDX) models and patient-derived xenograft (PDX) models.

CDX models are constructed by inoculating established common UM cell lines into the intraocular region or subcutaneously of immunodeficient mice (48, 49). Among them, intraocular inoculation (such as intrachoroidal injection) is closer to the occurrence scenario of human UM—the choroid is the most common primary site of UM, and this inoculation method can simulate the growth environment of tumors in the eye, allowing clear observation of the invasive process of tumors on ocular tissues; subcutaneous inoculation has the advantage of simple operation and is suitable for large-scale drug screening (48, 50, 51). The advantages of CDX models lie in their rapid construction, low cost, and strong reproducibility. Their limitation is that cell lines lose part of the genetic heterogeneity and microenvironmental characteristics of primary tumors during long-term culture, resulting in limited predictive value for treatment response.

PDX models are constructed by directly transplanting surgically resected UM tissues from patients into the intraocular region or under the renal capsule of immunodeficient mice, which can maximally retain the genetic heterogeneity, histopathological characteristics, and microenvironmental components of primary tumors (52, 53). The main limitations of PDX models are: difficulty in obtaining tumor tissues, low transplantation success rate (approximately 30%-50%), long construction cycle (usually 4–8 weeks), and the inability of immunodeficient mice to simulate the interaction between the human immune system and tumors, which restricts their application in immunotherapy research (54–56).

## 3 Animal models of desmoplastic melanoma

### 3.1 Overview of desmoplastic melanoma

DM exhibits a distinct genetic profile compared with common cutaneous melanoma. The mutation rate of *BRAF^V600E^* is only 5%–10%, much lower than that in conventional cutaneous melanoma; the mutation rate of *NRAS* is approximately 10%–15%, whereas inactivating mutations of the *NF1* gene are relatively common (about 20%–30%) (57–59).

In addition, DM is frequently associated with abnormal expression of genes related to the TGF-β signaling pathway, such as overexpression or activation of TGFB1, TGFBR1/2. These genetic alterations collectively drive malignant transformation of tumor cells and the formation of a fibrotic tumor microenvironment (60–62).

The fibrotic microenvironment is the most representative pathological feature of DM, mainly composed of activated cancer-associated fibroblasts (CAFs), abundant extracellular matrix (ECM) deposition, and scarce immune cells. As the central cellular component of the fibrotic microenvironment, CAFs secrete cytokines including TGF-β, platelet-derived growth factor (PDGF), and fibroblast growth factor (FGF), which further promote self-activation and ECM synthesis, forming a “fibrosis-amplifying loop” (63, 64). Dense ECM not only impedes drug penetration but also enhances the invasive capacity of tumor cells by activating integrin signaling, leading to an increased risk of local recurrence.

The immune microenvironment of DM displays a typical “cold tumor” phenotype: effector immune cells such as CD8^+^ T cells and NK cells are minimally infiltrated in tumor tissues, while the proportions of immunosuppressive cells including regulatory T cells (Tregs) and myeloid-derived suppressor cells (MDSCs) are elevated (65–67).

**Figure 2:**
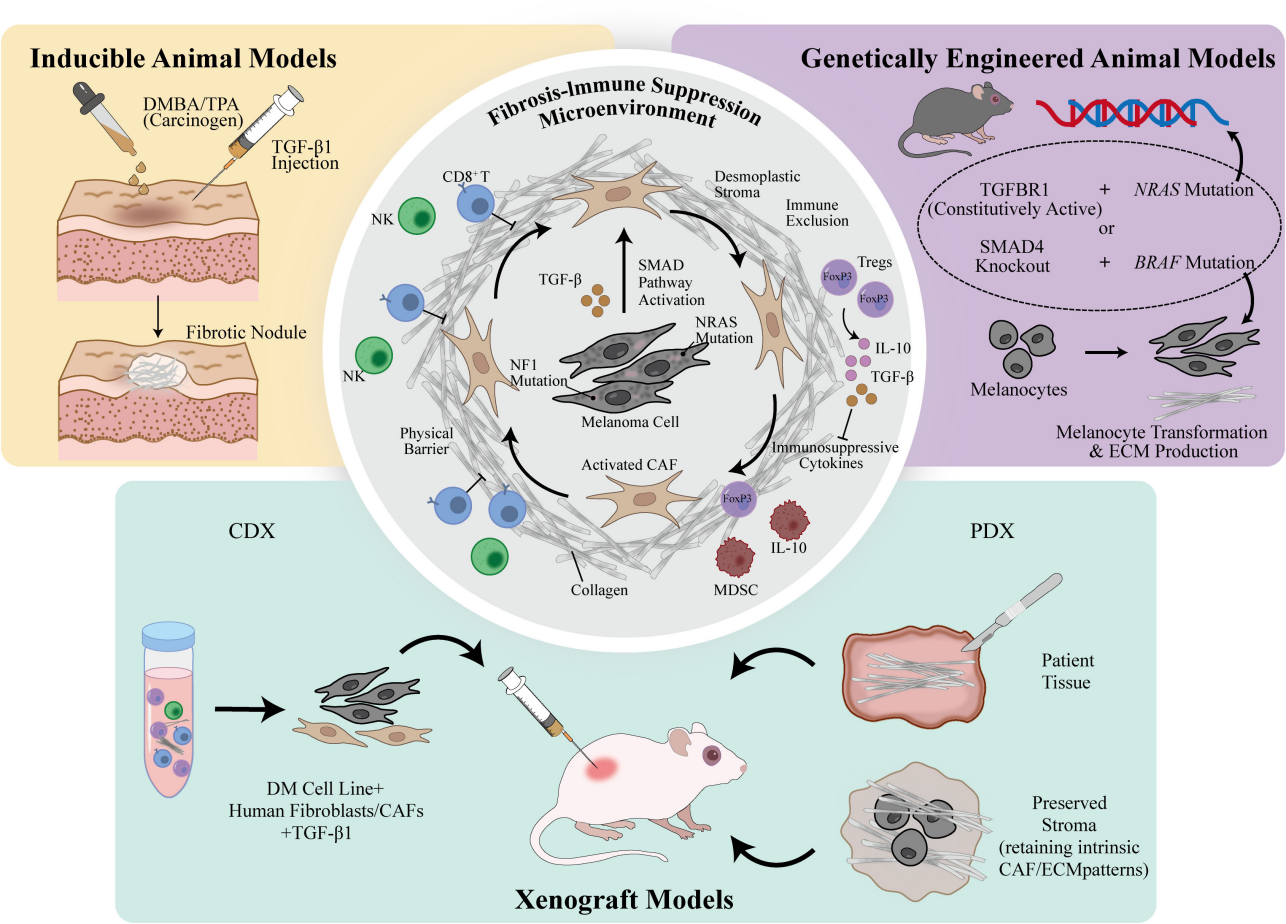
Pathogenesis and animal models of DM. The central mechanism highlights a fibrosis-immune suppression microenvironment, where melanoma cells harboring *NF1*/*NRAS* mutations secrete TGF-β to activate cancer-associated fibroblasts (CAFs) via the SMAD pathway. These activated CAFs generate a dense desmoplastic stroma rich in collagen, acting as a physical barrier that facilitates immune exclusion by blocking NK and CD8^+^ T cell infiltration. This immunosuppressive niche is further reinforced by the recruitment of FoxP3^+^ Tregs and MDSCs, which secrete cytokines like IL-10 and TGF-β. Surrounding this core mechanism are three distinct modeling strategies: Inducible Animal Models, established by applying the carcinogen DMBA/TPA alongside TGF-β1 injections to induce fibrotic nodules; Genetically Engineered Animal Models, which utilize specific genetic alterations (constitutively active *TGFBR1* with *NRAS* mutation, or *SMAD4* knockout with *BRAF* mutation) to drive melanocyte transformation and ECM production; and Xenograft Models, including CDX created by co-injecting DM cell lines with human fibroblasts/CAFs and TGF-β1, and PDX that preserve the intrinsic CAF/ECM patterns of patient tissue to faithfully recapitulate the tumor stroma.

Meanwhile, tumor cells and CAFs secrete immunosuppressive factors such as IL-10 and TGF-β, further suppressing anti-tumor immune responses. This immunosuppressive state accounts for the low response rate of DM to immune checkpoint inhibitors and represents an important target for developing combination therapeutic strategies (66, 68, 69) (**Figure 2**).

### 3.2 Inducible animal models

Inducible models generate melanoma with desmoplastic features in animals through physical, chemical or viral approaches. Featuring short construction cycles and low costs, they are suitable for large-scale screening studies.

Chemical induction is one of the most common methods. For example, topical application of 7,12-dimethylbenz[a]anthracene (DMBA) combined with 12-O-tetradecanoylphorbol-13-acetate (TPA) can induce melanoma in mouse skin, and some models present obvious fibrotic responses (70–73). Studies have found that local injection of TGF-β1 during induction can significantly enhance the fibrosis degree of tumor tissues, making the model more consistent with the pathological characteristics of DM (74, 75). Viral-mediated induction uses retroviruses to co-transfect oncogenes and TGFB1 into mouse melanocytes, which can rapidly establish melanoma models with typical fibrotic features (76, 77).

The defects of inducible models are the strong randomness of tumorigenesis, difficulty in accurately recapitulating the specific genetic background of human DM, and discrepancies between induced tumors and human DM in fibrosis degree and immune microenvironment characteristics. Thus, they are more suitable for macro-mechanistic studies of fibrosis formation rather than precise targeted therapy evaluation.

### 3.3 Genetically engineered animal models

Genetically engineered models accurately simulate the key genetic mutations and signaling pathway abnormalities of DM in animals via transgenesis, gene knockout or gene editing. With high genetic fidelity and strong reproducibility, they are the core models for current DM mechanistic research.

Targeting the core genetic and signaling pathway abnormalities of DM, two types of targeted genetically engineered models have been established, among which models based on the TGF-β signaling pathway are the research focus. Abnormal activation of the TGF-β signaling pathway is the core driver of fibrosis in DM. For instance, specific expression of mutant TGFBR1 (constitutively active form) in mouse melanocytes driven by the Tyrosinase tissue-specific promoter can induce melanoma with prominent desmoplastic features. In this model, collagen deposition in tumor tissues is significantly increased and CAFs are extensively activated, which is highly consistent with the pathological features of human DM (74, 75, 78). In addition, conditional knockout of Smad4 in mouse melanocytes combined with overexpression of *NRAS^V12^* can generate DM models with both malignant transformation and fibrotic phenotypes, which are used to study the synergistic mechanism of the TGF-β signaling pathway and oncogenes (79, 80).

The main limitations of genetically engineered models are as follows: the skin structure and immune environment of mice differ from those of humans, and the fibrosis degree of some models fails to reach the level of human DM; meanwhile, the long construction cycle, high technical threshold, and difficulty in simulating the complex genetic heterogeneity of DM limit their wide application (77, 81, 82).

### 3.4 Xenograft models

CDX models are constructed by inoculating established common DM cell lines subcutaneously or orthotopically (skin) in immunodeficient mice (82). To enhance the fibrotic phenotype, some studies co-inoculate human fibroblasts or locally inject TGF-β1 during tumor cell inoculation, which can significantly promote fibrosis in tumor tissues. For example, co-inoculation of WM239A cells and human dermal fibroblasts subcutaneously in nude mice results in markedly increased collagen deposition and upregulated expression of CAF activation markers in tumor tissues, which better mimics the pathological features of DM (81–83). This model is easy to operate and quick to construct, suitable for large-scale screening of drugs targeting fibrosis or tumor cells. Its shortcomings include the loss of partial genetic heterogeneity and fibrotic microenvironment characteristics of primary tumors during long-term cell line culture, and the inability of immunodeficient mice to simulate the interaction between the host immune system and tumors, leading to limited value for immunotherapy evaluation (83, 84).

PDX models are established by directly transplanting surgically resected DM tissues from patients subcutaneously or orthotopically in immunodeficient mice, which can maximally preserve the genetic heterogeneity, histopathological features and fibrotic microenvironment of primary tumors (81, 82). Studies have shown that DM PDX models are highly consistent with primary tumors in tumor tissue morphology, CAF activation status and collagen deposition pattern, and can be stably passaged, serving as the “gold standard” for evaluating personalized therapeutic strategies among xenograft models (85, 86). The main limitations of this model are: difficulty in obtaining tumor tissues, low transplantation success rate of DM-specific PDX models (approximately 25%-40%), long construction cycle, and reliance on immunodeficient mice that cannot recapitulate the real efficacy of immunotherapies, restricting its application in immunotherapy research (20, 87).

## 4 Animal models of mucosal melanoma

### 4.1 Overview of mucosal melanoma

MM accounts for approximately 1.3%-3.8% of all melanoma cases and predominantly occurs in mucosal sites including the digestive tract, genitourinary tract, and respiratory tract, among which the oral cavity, nasal cavity and paranasal sinuses, as well as rectum and anal canal are the most common origins (20, 88).

Different from cutaneous melanoma, the development of MM is unrelated to ultraviolet exposure, and it exhibits highly specific genetic driver characteristics: the mutation rate of the *C-KIT* gene is as high as 13.5%-35.5%, mutations in *SF3B1*, *BAP1* and other genes are also relatively common, whereas the mutation rate of *BRAF^V600E^* is only 5%-10%, much lower than that in cutaneous melanoma (89–91).

Clinical data demonstrate that most MM patients are diagnosed at intermediate or advanced stages, with extremely high rates of local recurrence and distant metastasis. Distant metastasis mainly affects the liver, lungs, and brain, and the 5-year survival rate of patients is merely 25%-30%. The response rate of MM to conventional chemotherapy and immune checkpoint inhibitors is significantly lower than that of cutaneous melanoma (92, 93).

A core biological feature of MM is its intimate interaction with the mucosal microenvironment. The immunosuppressive microenvironment beneath the mucosal epithelium, characterized by massive infiltration of regulatory T cells and macrophages and secretion of immunosuppressive factors such as TGF-β and IL-10, provides favorable conditions for malignant transformation and immune escape of tumor cells (92, 94, 95).

In addition, MM from different sites possesses unique organ-specific microenvironmental characteristics, such as the crosstalk between digestive tract MM and gut microbiota, and the association between genitourinary tract MM and the hormonal microenvironment. Emerging evidence suggests that gut microbiota can modulate mucosal immune responses, inflammatory signaling, and antitumor immunity, thereby potentially influencing MM progression and therapeutic responses. However, the precise mechanisms underlying microbiome–tumor interactions in MM remain poorly understood and warrant further investigation. These features collectively determine the pathogenesis and therapeutic response heterogeneity of MM (92, 96, 97).

**Figure 3:**
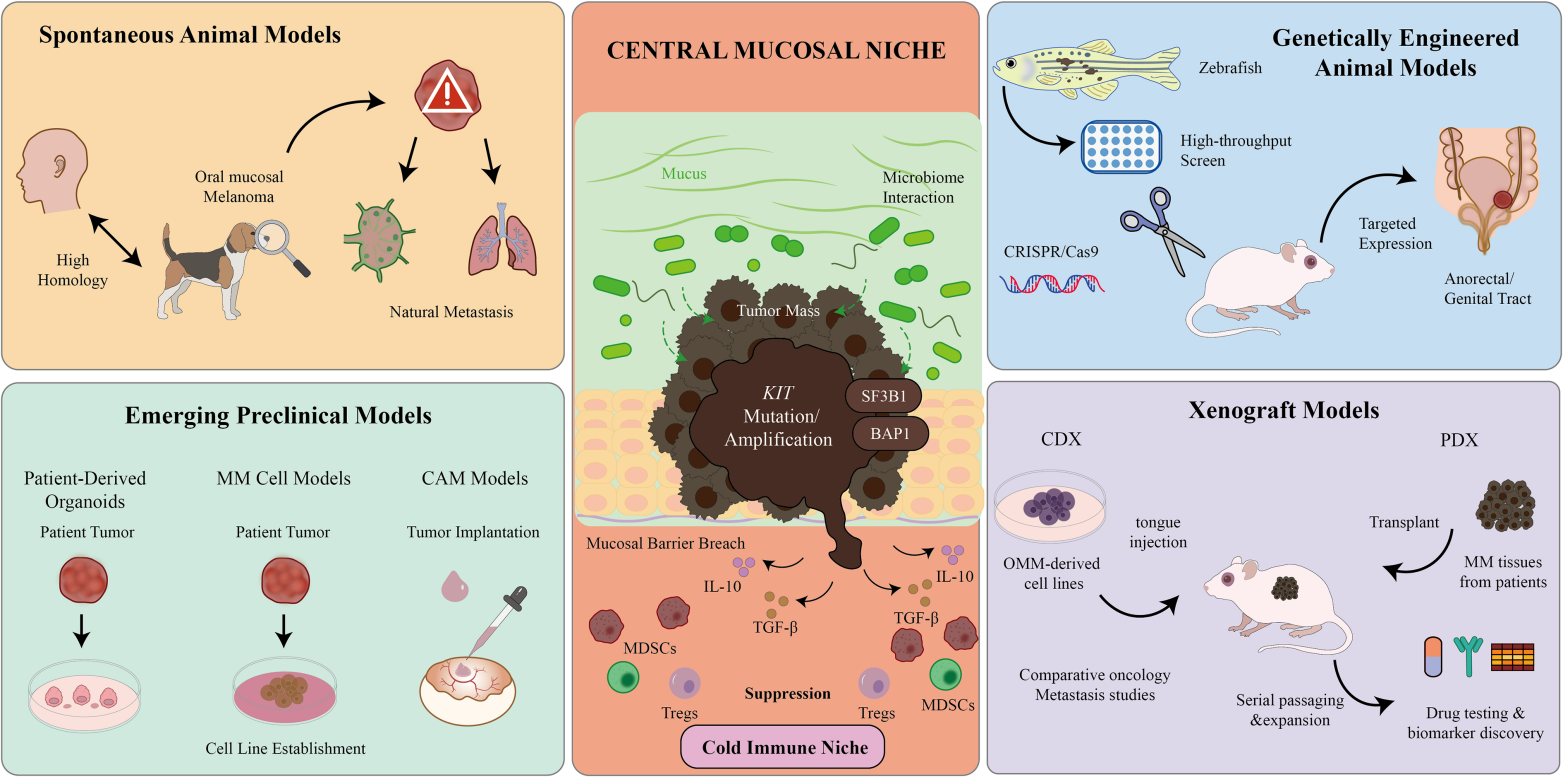
Representative animal and preclinical models of mucosal melanoma and their associated microenvironmental features. Spontaneous animal models, particularly canine oral mucosal melanoma with high homology to human disease, recapitulate natural metastasis and provide valuable systems for studying tumor initiation and progression. The central mucosal niche is characterized by mucus and microbiome interactions, recurrent genomic alterations including KIT mutation/amplification, SF3B1, and BAP1 abnormalities, and an immunosuppressive cold immune niche formed after mucosal barrier breach, with release of IL-10 and TGF-beta and recruitment of MDSCs and Tregs. Genetically engineered animal models combine high-throughput screening in zebrafish with CRISPR/Cas9-based targeted expression in mice to model anorectal and genital tract mucosal melanoma. Emerging preclinical models include patient-derived organoids, mucosal melanoma cell models established from patient tumors, and CAM models generated by tumor implantation. Xenograft models include CDX systems established by tongue injection of oral mucosal melanoma-derived cell lines for comparative oncology and metastasis studies, and PDX models generated by transplantation of patient-derived mucosal melanoma tissues for serial passaging, drug testing, and biomarker discovery.

However, due to the concealed anatomical location, scarcity of clinical samples, and complexity of the mucosal microenvironment, it is difficult to fully elucidate the pathophysiological mechanisms of MM relying solely on clinical studies, highlighting the urgent demand for reliable animal models as research tools (**Figure 3**).

### 4.2 Spontaneous models

Spontaneous models refer to tumor models that develop in animals driven by natural mutations or genetic background, which most closely recapitulate the natural occurrence and progression of human diseases.

The representative model of this category is the canine oral MM model, which also serves as one of the important gold standards in the research of MM (98). Its core characteristics and advantages are reflected in the high similarity between canine MM and human MM in terms of histopathological features, molecular expression profiles, high metastatic tendency, and responsiveness to immunotherapy (99, 100). Moreover, the tumors arise spontaneously in the context of an intact host immune system, which enables the complete preservation of the complex crosstalk between tumors and the host microenvironment, conferring irreplaceable clinical relevance to this model (101, 102). Histopathologically, canine OMM closely resembles human MM, exhibiting similar invasive growth patterns and expression of melanocytic markers including Melan-A, PNL2, and S100, supporting its translational relevance (98, 100).

This model is mainly used to elucidate the natural pathogenic process of MM, explore the molecular mechanisms of tumor metastasis, and evaluate the *in vivo* efficacy of novel immunotherapeutic regimens. Its limitations mainly include the high feeding cost of experimental animals, long research cycles, and individual genetic background variations among animals that may affect the consistency of experimental results (103).

### 4.3 Genetically engineered models

Genetically Engineered Models refer to tumor models that drive melanoma initiation in a targeted manner by introducing exogenous oncogenes into animals or precisely editing their endogenous genes via genetic engineering technologies such as transgenesis and gene editing (104).

It is mainly divided into two types: First, transgenic models, in which expression vectors carrying specific oncogenes are constructed to achieve the specific expression of these genes in murine melanocytes, thereby driving melanoma development (105, 106). Second, *in situ* gene-edited models; for instance, editing neural crest-related genes in zebrafish models using technologies such as CRISPR-Cas9 can induce melanoma with the characteristics of high metastasis and low immunogenicity, and the gene expression profile of tumors in this model is highly similar to that of human MM (107, 108).

The core advantages of such models are the ability to precisely elucidate the biological functions of specific driver genes and clarify their molecular regulatory mechanisms in the occurrence and development of MM. Among them, the zebrafish model also has the unique advantage of *in vivo* real-time imaging, which enables the dynamic observation of the invasive and metastatic behaviors of tumor cells *in vivo (*108, 109). It is mainly used to explore the genetic basis of MM and the regulatory mechanisms of key signaling pathways. Its limitations include the complex construction process and high experimental cost of transgenic mouse models, and the modification of a single gene cannot fully simulate the genetic background of multi-gene synergistic mutations in human MM (110–112).

### 4.4 Xenograft models

PDX models have emerged as essential tools for MM, a rare subtype with distinct genomic features and low mutational burden compared with cutaneous melanoma (6, 113). Due to the scarcity of established cell lines and patient samples, MM PDX models provide a valuable platform to investigate tumor biology and evaluate therapeutic responses. In addition to human MM PDXs, canine oral mucosal melanoma (OMM)-derived cell lines and xenogeneic mouse models have recently been established and shown to retain key malignant characteristics of the parental tumors, including tumorigenicity and metastatic potential. Given the substantial clinical and molecular similarities between canine OMM and human MM, these models provide a complementary comparative oncology platform for mechanistic studies and preclinical therapeutic evaluation (98, 114). Notably, integrated genomic and PDX analyses have demonstrated that MM PDXs preserve the molecular characteristics of the original tumors and can be used to identify therapeutically actionable alterations, including CDK4 amplification-associated sensitivity to palbociclib (113). In addition, large melanoma PDX repositories incorporating mucosal melanoma specimens have enabled studies of tumor heterogeneity, drug resistance, and biomarker discovery across melanoma subtypes (86, 115). To improve accessibility, the Melanoma Research Alliance (MRA) has established a dedicated mucosal melanoma model catalog that facilitates model sharing and accelerates translational research efforts (Melanoma Research Alliance, https://www.curemelanoma.org/research/researcher-resources/mucosal-melanoma-model-catalog).

Despite their utility, MM PDX models possess several inherent limitations. As with other xenograft systems, human stromal components are progressively replaced by murine counterparts during serial passaging, while the requirement for immunodeficient hosts precludes the study of native tumor–immune interactions (116). Furthermore, long-term propagation may introduce clonal selection and genetic divergence from the original patient tumor, potentially affecting therapeutic response assessments (86, 116). Similarly, although canine OMM-derived xenografts provide a valuable comparative oncology platform, species-specific genetic differences and the relatively limited availability of well-characterized canine melanoma resources may constrain their direct translational applicability to human disease. Consequently, MM PDX models are best utilized alongside humanized mouse models and complementary *in vitro* systems to provide a more comprehensive representation of tumor biology and treatment response. In addition, most currently available MM PDX and CDX resources are derived from oral or head-and-neck mucosal melanomas, whereas models representing gastrointestinal, anorectal, and genitourinary MM remain limited, potentially introducing anatomical-site bias and restricting the generalizability of experimental findings (6, 117).

### 4.5 Emerging preclinical models

Recent advances have expanded the repertoire of preclinical models available for MM, particularly through the development of patient-derived organoids (PDOs), advanced cellular systems, and chick chorioallantoic membrane (CAM) models (16). PDOs preserve key histopathological and molecular features of the original tumors and can recapitulate inter- and intrapatient heterogeneity, making them valuable tools for drug screening and precision medicine studies (118, 119). Notably, OMM organoids have been used to investigate immunotherapy responses and mechanisms of resistance, including NGFR-associated signaling pathways (118).

In addition, recently established patient-derived MM cell models have facilitated mechanistic studies and therapeutic evaluation. These systems retain important molecular characteristics of MM and have contributed to the identification of melanoma-initiating cell populations and signaling pathways involved in tumor maintenance, such as PI3K/Akt/mTOR signaling (120). The development of these models has been further supported by genomic studies demonstrating that MM harbors distinct molecular alterations compared with cutaneous melanoma, emphasizing the need for subtype-specific experimental platforms (6, 117). Meanwhile, CAM models provide a rapid and cost-effective *in vivo* platform for assessing tumor growth, angiogenesis, invasion, and preliminary therapeutic responses, serving as a useful complement to conventional murine models (16, 121).

Despite these advantages, important limitations remain. Organoids and cell models cannot fully reproduce the complex tumor microenvironment, including immune, stromal, and vascular interactions, and prolonged culture may introduce clonal selection or genetic drift (119, 122). Likewise, the short experimental duration and immature immune system of CAM models restrict their utility for studying long-term tumor evolution and immunotherapeutic responses (16, 121). Therefore, these emerging platforms are best viewed as complementary tools that can be integrated with established GEMM, PDX, and comparative oncology models to improve translational MM research (123).

## 5 Animal models of acral melanoma

### 5.1 Overview of acral melanoma

AM accounts for more than 60% of melanoma cases in Asian populations, compared with only 1%–3% in European and North American populations, making it the most prevalent melanoma subtype among non-Caucasian populations (124). Unlike conventional cutaneous melanoma, AM is largely unrelated to UV exposure and exhibits distinct clinicopathological and molecular characteristics (125).

Clinically, most patients are diagnosed at intermediate or advanced stages owing to the concealed anatomical locations of lesions and the lack of early symptoms (126, 127). Following progression from acral melanoma *in situ* (AMis) to invasive acral melanoma (iAM), the risk of distant metastasis increases substantially, with the lung, liver, and brain being the most common metastatic sites (128). Consequently, the 5-year survival rate remains only 40%–60%, significantly lower than that observed in other cutaneous melanoma subtypes (125, 129).

The biological characteristics of AM can be summarized into three major aspects. First, AM possesses a distinct genomic landscape. The prevalence of *BRAF^V600E^* mutations is relatively low (approximately 14.5%), whereas alterations involving *KIT, NRAS, NF1*, and other driver genes are more frequently observed, often accompanied by extensive copy-number alterations and chromosomal instability (130, 131). Recent genomic profiling studies have further expanded the spectrum of potentially actionable molecular alterations in AM (132).

Second, the tumor immune microenvironment exhibits a pronounced immunosuppressive phenotype. Sparse infiltration of CD8^+^ T cells is frequently accompanied by enrichment of APOE^+^/CD163^+^ macrophages, which facilitate epithelial–mesenchymal transition (EMT) through activation of the IGF1/IGF1R signaling axis (133). In addition, aberrant expression of multiple immune checkpoint molecules contributes to immune evasion and therapeutic resistance (133, 134).

Third, AM develops within a unique anatomical and biomechanical niche. Acral sites are characterized by glabrous skin, a thick stratum corneum, dense neurovascular networks, and chronic mechanical stress (135, 136). More importantly, recent evidence has demonstrated that melanocytes from distinct anatomical locations possess site-specific transcriptional programs that influence susceptibility to particular oncogenic events and shape melanoma subtype identity (137, 138). These findings suggest that anatomical context may play a role comparable to genetic alterations in determining melanoma subtype identity and may partially explain why conventional melanoma models often fail to faithfully recapitulate human AM.

**Figure 4:**
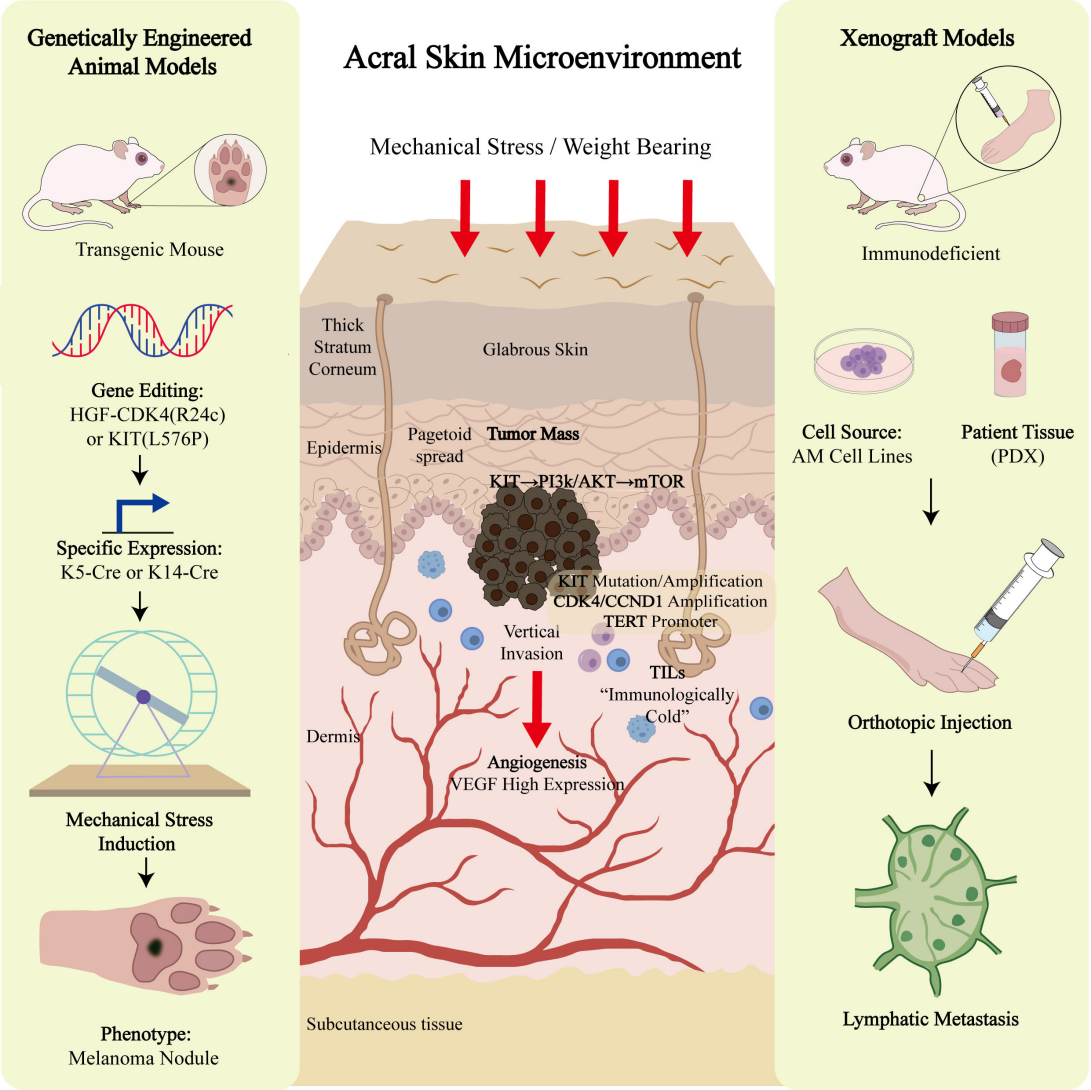
Pathogenesis and animal modeling strategies for AM. The central illustration depicts the Acral Skin Microenvironment, characterized by glabrous skin with a thick stratum corneum subject to mechanical stress and weight-bearing. Within this niche, tumor masses harboring key genetic alterations—specifically *KIT* mutations/amplifications driving the PI3K/AKT/mTOR pathway, alongside CDK4/CCND1 amplifications and TERT promoter mutations—exhibit pagetoid spread and vertical invasion into the dermis and subcutaneous tissue. The microenvironment is portrayed as “immunologically cold” with sparse Tumor-Infiltrating Lymphocytes (TILs) but high angiogenic activity mediated by elevated VEGF expression. Surrounding this central mechanism are two modeling approaches: Genetically Engineered Animal Models, which utilize transgenic mice with specific gene edits (e.g., *HGF*-*CDK4*(*R24c*) or *KIT*(*L576P*)) driven by K5-Cre or K14-Cre promoters, often combined with mechanical stress induction to generate melanoma nodules; Xenograft Models, employing immunodeficient mice to receive orthotopic injections of AM cell lines or patient-derived tissue to mimic lymphatic metastasis.

Therefore, systematic evaluation of existing AM animal models and careful assessment of their strengths and limitations are essential for advancing mechanistic studies and accelerating the development of effective therapeutic strategies for this aggressive melanoma subtype (**Figure 4**).

### 5.2 Genetically engineered models relevant to acral melanoma

Genetically engineered animal models have been widely used to investigate molecular alterations associated with AM. However, most currently available systems reproduce selected oncogenic events rather than the complete anatomical, genomic, and microenvironmental characteristics of human AM (104, 137).

To date, no genetically engineered model fully recapitulates the defining features of human AM. Most available systems should therefore be regarded as models of AM-associated oncogenic pathways rather than bona fide AM models (104).

Recent studies have highlighted the importance of anatomical context in melanoma initiation and subtype specification. Weiss et al. demonstrated that melanocytes residing at different anatomical locations possess distinct transcriptional programs that influence susceptibility to specific oncogenic events (137). Supported by subsequent analyses and commentary, these findings indicate that anatomical position itself contributes to melanoma subtype identity (138). Consequently, introduction of melanoma driver mutations alone may be insufficient to generate authentic AM.

Several genetically engineered mouse models have been employed to investigate genetic alterations commonly observed in AM. Burd et al. demonstrated that oncogenic *NRAS* variants exhibit mutation-specific tumorigenic potential, with NRASQ61R showing stronger melanomagenic activity than other *NRAS* mutations (139). Similarly, melanocyte-targeted delivery of NRASQ61R combined with Ink4a/Arf loss effectively induces melanoma formation in mice (140). Although these models provide valuable insight into NRAS-driven melanomagenesis, they were not designed as AM-specific systems and do not reproduce the characteristic anatomical features of human AM (139, 140).

Additional multi-hit models incorporating activated NRAS signaling, CDK4R24C activation, or loss of keratinocytic RXRα have further demonstrated cooperative interactions among oncogenic pathways during melanoma development (141). Nevertheless, several important limitations should be acknowledged. First, CDK4R24C represents constitutive activation of CDK4 rather than the high-level amplification of wild-type CDK4 frequently observed in human AM (132, 141). Second, UVB irradiation is required to promote tumor formation in these models, whereas AM generally lacks the UV-associated mutational signatures characteristic of conventional cutaneous melanoma (125, 132, 141). Third, melanomas typically arise on dorsal skin rather than bona fide acral sites (141). Therefore, although these systems are useful for investigating melanoma initiation and cooperative oncogenic signaling, they do not fully recapitulate human AM biology.

In zebrafish, studies exploring anatomical transcriptional programs and melanoma-associated signaling pathways have provided important mechanistic insights into melanoma subtype specification and tumor development (137, 138). These models offer advantages including rapid genetic manipulation, optical transparency, and high-throughput screening capability. However, similar to current murine systems, they primarily model molecular pathways associated with AM rather than reproducing the full anatomical and microenvironmental complexity of human disease.

Collectively, existing genetically engineered systems are best regarded as models for studying AM-associated oncogenic mechanisms rather than faithful representations of human AM. Future efforts should integrate AM-specific genomic alterations, spatially restricted gene engineering strategies, and acral microenvironmental features to improve disease fidelity (104, 132, 137).

### 5.3 Xenograft models

Xenograft models remain important translational platforms for investigating tumor progression and therapeutic responses in AM (82, 142).

CDX models are commonly established through implantation of melanoma cells into immunodeficient mice. Several studies have employed orthotopic footpad implantation approaches to mimic the anatomical location of acral lesions and facilitate investigation of local invasion, lymphatic dissemination, and metastatic progression (142, 143). However, implantation into an acral site does not inherently generate an acral melanoma model. Commonly used murine melanoma cell lines such as B16-F1 and B16-F10 originated from spontaneous cutaneous melanomas rather than bona fide acral melanomas (142, 143). Therefore, footpad inoculation primarily recreates tumor growth within an acral anatomical location and should not be considered a true AM model.

To better mimic the tumor microenvironment, co-culture and co-transplantation approaches incorporating fibroblasts and stromal components have been developed to evaluate stromal contributions to melanoma invasion and metastasis (144). These strategies provide additional insight into tumor–stroma interactions but still cannot fully reproduce the complex biological characteristics of human AM.

PDX models currently provide the highest level of biological fidelity among xenograft approaches. Direct transplantation of patient-derived AM tissues preserves tumor heterogeneity, genomic architecture, histopathological characteristics, and treatment-associated molecular features (142, 145). Establishment of primary acral melanoma cultures and corresponding xenografts has enabled preclinical evaluation of targeted therapies and investigation of tumor–microenvironment interactions (142). Furthermore, recent studies demonstrated that receptor tyrosine kinase inhibition can induce regression of AM xenografts through modulation of the tumor microenvironment, highlighting the translational value of these systems for therapeutic development (146).

Despite these advantages, PDX models remain constrained by limited tissue availability, relatively low engraftment rates, prolonged establishment times, and the absence of a fully functional immune system in recipient mice (82, 145). Consequently, xenograft models should be viewed as complementary tools for translational and therapeutic studies rather than definitive representations of AM pathogenesis.

## 6 Future perspectives and outlook

### 6.1 Future directions in rare melanoma modeling

Due to the specificity of genetic background, microenvironmental characteristics, and clinical behavior among different subtypes of rare melanoma, the construction of their animal models still faces many challenges. With the rapid development of biotechnology, future model construction will advance toward high-fidelity, multi-dimensional simulation, and clinically translatable platforms.

In terms of model optimization, gene-editing technologies, particularly spatiotemporally controlled CRISPR-Cas9-based approaches, are expected to further improve the accuracy of genetic simulation and enable the targeted introduction of cooperative multi-gene alterations that more faithfully recapitulate tumor heterogeneity. In parallel, advances in tissue engineering, biomaterial-based three-dimensional culture systems, and microfluidic organ-on-chip technologies have provided new opportunities to reconstruct physiologically relevant tumor microenvironments and to model complex tumor–host interactions. These platforms enable precise control of biomechanical cues, extracellular matrix composition, fluid flow, immune cell trafficking, and tissue-specific signaling, thereby overcoming several limitations of conventional *in vitro* and *in vivo* models (147–149).

For rare melanoma research, the integration of tissue engineering and microfluidic technologies may facilitate the reconstruction of subtype-specific microenvironments, such as ocular immune privilege in uveal melanoma, chronic mechanical stress in acral melanoma, and host–microbiota interactions in mucosal melanoma (147–149). Furthermore, optimization of humanized models will increasingly focus on the coordinated reconstruction of both the immune system and organ microenvironment, thereby improving immune cell reconstitution, functional fidelity, and translational relevance for mechanistic studies and immunotherapeutic evaluation.

Beyond the optimization of existing animal models, emerging model organisms may further broaden the experimental landscape for melanoma research. Among them, Xenopus has attracted increasing attention owing to its distinctive cutaneous pigment cell biology, optical accessibility during development, and amenability to genetic manipulation. These characteristics make Xenopus a promising complementary platform for investigating melanocyte biology, melanoma initiation, tumor–microenvironment interactions, and therapeutic responses. As molecular engineering and imaging technologies continue to advance, Xenopus is expected to further contribute to mechanistic studies and translational research in melanoma (150–152).

### 6.2 Model selection considerations

**Figure 5:**
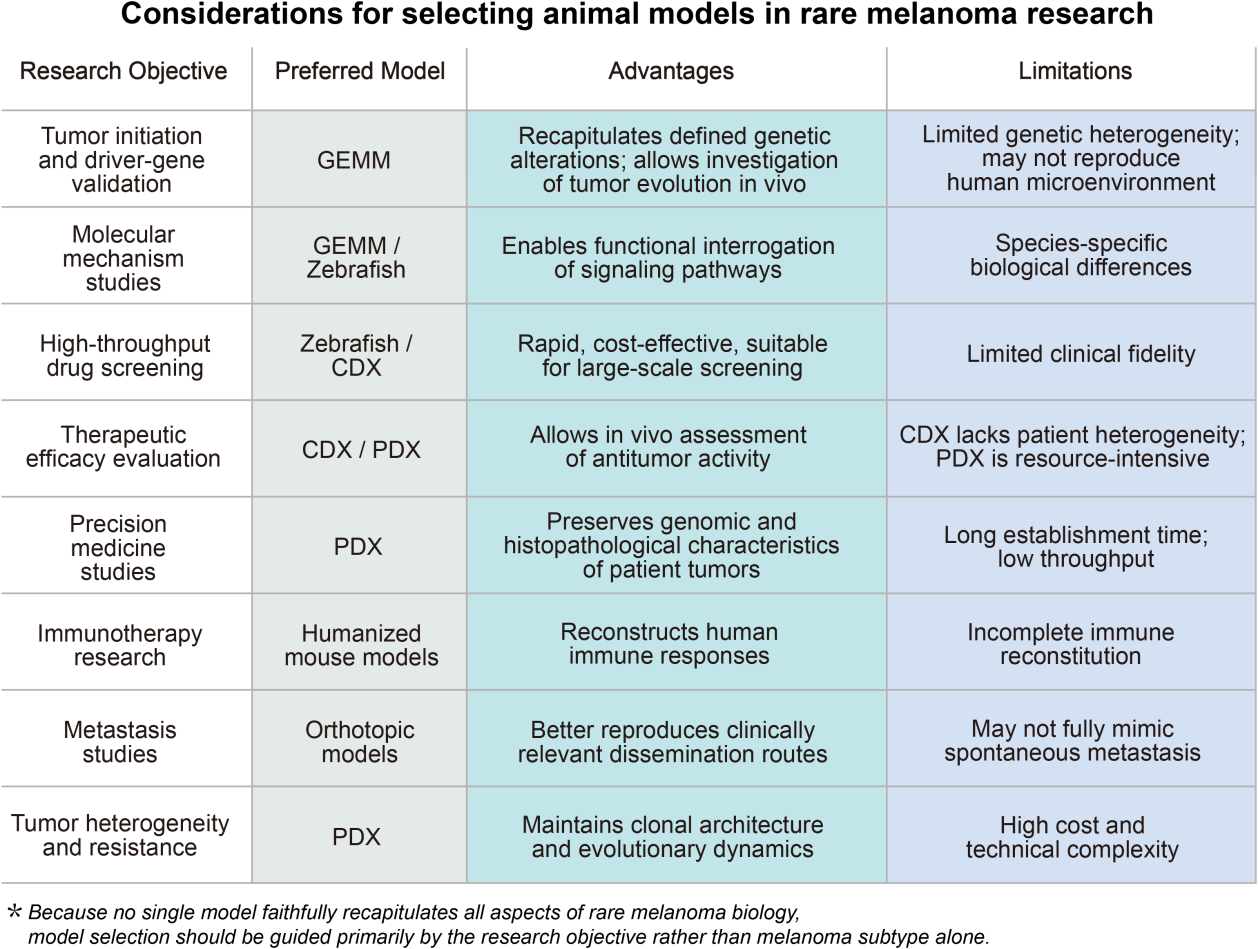
Different animal models offer distinct advantages for addressing specific research questions in rare melanoma. Genetically engineered mouse models (GEMMs) are particularly useful for investigating tumor initiation and driver-gene function, whereas cell-derived xenograft (CDX) and zebrafish models provide efficient platforms for drug screening. Patient-derived xenograft (PDX) models preserve patient-specific tumor characteristics and are therefore valuable for precision medicine and studies of therapeutic resistance. Humanized mouse models enable evaluation of immune–tumor interactions and immunotherapies, while orthotopic models are advantageous for investigating metastatic behavior. Because each model has inherent limitations, complementary use of multiple model systems is often required to maximize translational relevance.

Given the substantial biological heterogeneity among rare melanoma subtypes and the diverse objectives of preclinical research, no single animal model can adequately address all experimental needs. Therefore, the selection of an appropriate model should be guided primarily by the specific research question rather than by melanoma subtype alone. Different model systems possess distinct strengths and limitations in studies of tumor initiation, molecular mechanisms, drug development, immunotherapy, metastasis, and precision medicine. To facilitate model selection, the major applications, advantages, and limitations of currently available rare melanoma models are summarized in **Figure 5**.

## 7 Conclusion

Animal models of rare melanoma are essential tools for dissecting disease mechanisms, screening therapeutic targets, and evaluating novel therapies. This review systematically summarizes the research progress of animal models for UM, DM, MM, and AM, covering four major categories: spontaneous, inducible, genetically engineered, and xenograft models.

Each type of model has distinct advantages and limitations. Spontaneous models preserve the complete process of natural tumorigenesis and host interaction, suitable for clinical translational verification, but suffer from long cycles and poor controllability. Inducible models are convenient to construct and low-cost, appropriate for macro-mechanistic exploration, yet lack precision in genetic simulation. Genetically engineered models feature high genetic fidelity and strong reproducibility, serving as the core for mechanistic research, but fail to fully recapitulate complex microenvironments. Xenograft models (especially PDX models) retain the characteristics of primary tumors and support personalized therapy, but the immunodeficient background limits immunotherapy-related research. Humanized models can simulate the interaction between the human immune system and microenvironment, but involve complex techniques and high costs.

In the future, it is necessary to integrate multi-technological strategies to optimize model construction according to the specific requirements of each subtype, and improve the dual fidelity of genetic simulation and microenvironment recapitulation. Meanwhile, strengthening the correlation and validation between models and clinical samples will promote the translation of research findings into clinical practice, providing a solid experimental foundation for mechanistic breakthroughs and therapeutic innovations of rare melanoma.
